# Photocatalytic hydrogen generation of monolithic porous titanium oxide-based glass–ceramics

**DOI:** 10.1038/s41598-020-68410-7

**Published:** 2020-07-15

**Authors:** Hirokazu Masai, Hiroaki Sakurai, Akitoshi Koreeda, Yasuhiro Fujii, Takahiro Ohkubo, Takamichi Miyazaki, Tomoko Akai

**Affiliations:** 1 National Institute of Advanced Industrial Science and Technology, 1-8-31 Midorigaoka, Ikeda, Osaka 563-8577 Japan; 2 Department of Physical Sciences, Ritsumeikan University, 1-1-1 Nojihigashi, Kusatsu, Shiga 525-8577 Japan; 3 Graduate School & Faculty of Engineering, Chiba University, 1-33, Yayoi-cho, Chiba, 263-8522 Japan; 4 Technical Division, Graduate School of Engineering, Tohoku University, 6-6-11, Aoba, Sendai 980-8579 Japan

**Keywords:** Structure of solids and liquids, Glasses, Photocatalysis, Porous materials

## Abstract

A large relative surface area is crucial for high catalytic activity. Monolithic catalysts are important catalytic materials because of minimal self-degradation. Regarding large surface area catalysts, the glass–ceramics (GCs) with high formability, obtained by heat-treatment of the precursor glass, are plausible candidates. This study examines the photocatalytic behaviour of porous GCs obtained after acid leaching of MgO–TiO_2_–P_2_O_5_ GCs. After heat-treatment, anatase TiO_2_ was precipitated along with other phases. The diffraction intensity ratio between anatase and other phases was the maximum for a heat-treatment temperature of 900 °C. After acid leaching of the GCs, the relative surface area decreased with increasing TiO_2_ fraction; the surface area was also affected by the sample morphology. H_2_ generation was observed from porous GCs, while GCs without etching exhibited approximately zero activity. Thus, it was demonstrated that high surface area and prevention of the reduction reaction to Ti(III) are important for tailoring monolithic photocatalytic materials.

## Introduction

The development of photocatalytic activity without any energy consumption is a major issue in energy harvesting. Use of stable photocatalytic materials using sunlight is one solution. Considering the relative surface area of the sea, water can be a resource for energy harvesting. However, in the case of powdered catalytic materials in a liquid, precipitation of the crystallites at the bottom of the system is inherently unavoidable without stirring, i.e., some energy introduction is required for continuous catalytic activity of the powdered materials in the liquid. Considering spontaneous energy conversion using water, bulk shapes, which can be placed on the surface of water bodies such as seas or lakes, are needed. Therefore, the focus here is on porous bulk photocatalytic materials.

TiO_2_ is a major oxide semiconductor that possesses high chemical stability and photocatalytic activity. The photocatalytic activity of TiO_2_-related materials has been enthusiastically examined the world over. The studies were predominantly on powdered materials, as relative surface area is a dominant factor in achieving high catalytic activity. However, TiO_2_ nanofiber, which is a nanostructured TiO_2_, has been recently proposed, in addition to TiO_2_ powders^1–17^. Although it was reported that a pure porous TiO_2_ monolith was demonstrated via the sol–gel method^18^, it is tedious to fabricate materials of large surface area using such nanostructured materials.

One approach to fabricating large porous TiO_2_ materials is to use a glass–ceramics (GC) route combined with chemical etching^19–24^. As glass is in a thermodynamically metastable state at a certain temperature, crystallization occurs above the glass transition temperature *T*_g_. Although the crystallization of glass is affected by several factors, such as chemical composition of the mother glass and the heat-treatment temperature, the glass–ceramics-containing preferred crystallites can possess the characteristics of both glass and crystal. As TiO_2_ possesses a high refractive index and a high nucleating ability during the heat-treatment of glass, it has been used as a key component in oxide glasses, especially in GCs^25,26^. However, precipitation of TiO_2_ crystallites as a precipitated phase is rare. If the fraction of TiO_2_ increases, other crystallites are often observed^27,28^. However, the volume fraction of TiO_2_-precipitated GCs with TiO_2_ precipitated as a single phase^29–31^ is not sufficient to make a porous skeleton network.

Hosono et al. reported several functional crystallites, such as anatase TiO_2_, NASICON-type CaTi_4_(PO_3_)_4_^22^ and CaTi_4_(PO_4_)_6_^23^ from Na-doped CaO–TiO_2_–P_2_O_5_ glasses^19^. In these reports, various applications using the porous structure and the constituent cations were discussed^19^. Although some TiO_2_–SiO_2_ porous materials were obtained^23,24^, these multi-component materials were either phase-separated or not amorphous after quenching, and, therefore, were not suitable for the design of TiO_2_ precipitation. On the contrary, as Ca^2+^ and Mg^2+^ play a similar role in the vitrification of glass, it is expected that TiO_2_-precipitated GCs can also be obtained from MgO-TiO_2_-P_2_O_5_ (MTP) ternary glasses. However, there are relatively few reports on ternary MTP glasses^32^. MTP glasses have been prepared with the glass forming region, and their density and the molar volume have been reported by Kishioka^32^. Although it was reported that the minimum P_2_O_5_ and maximum TiO_2_ fractions in the MTP glasses without precipitation of the crystallites were 30 mol.% and 35 mol.%, respectively, there was no detailed information about the precipitated phase and the thermal stability^32^. As vitrification of glass using a melt-quenching method depends on its mode of preparation, it is expected that broader chemical compositions of MTP glass can be obtained by tuning the quenching process.

In the present study, the focus was on porous TiO_2_-containing GCs using the MTP glass system via the GC route. In addition to conventional X-ray diffraction (XRD) and scanning electron microscope (SEM) technique, the structural change was examined, depending on chemical composition, using X-ray absorption fine structure (XAFS), ^31^P magic angle spinning (MAS) NMR, and elastic modulus measurements. For establishing the guidelines for photocatalytic hydrogen generation of monolithic materials, physical parameters and structure of porous GCs have been examined using a combination of various analytical methods in addition to photocatalytic activities.

## Results and discussion

**Figure 1:**
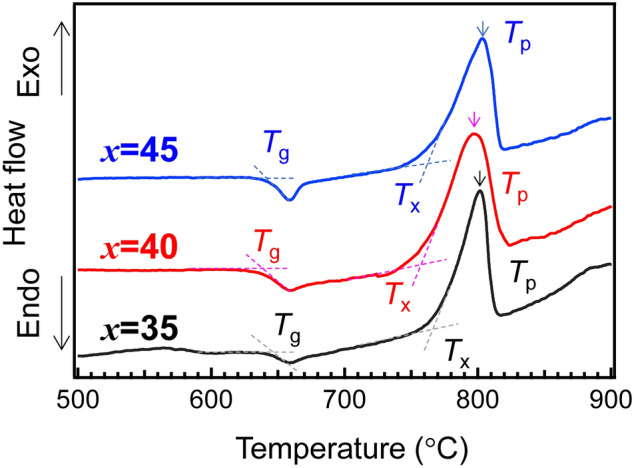
DTA curves of MTP*x* glasses: *T*_g_, *T*_x_, and *T*_p_ indicate the glass transition temperature, temperatures of crystallization onset, and crystallization peak, respectively.

Figure 1 shows the differential thermal analysis (DTA) curves of (70-*x*) MgO–*x*TiO_2_–30P_2_O_5_ (MTP*x*) glasses. These DTA curves were similar and independent of the TiO_2_ fraction. The temperature of glass transition, *T*_g_, of these glasses was approximately 640 °C, whose error bars were comparable to those of the DTA measurements. The temperatures of crystallization onset, *T*_*x*_ and the crystallization peak *T*_*p*_ are also shown in Fig. 1. Considering the thermal stability of the glasses was estimated using the Δ*T* (= *T*_x_ − *T*_g_) values^33^, the MTP40 glass was highly unstable against crystallization. However, as the *T*_g_ and the *T*_p_ were similar, it was assumed that same heat-temperature was suitable for comparison of each sample.

**Figure 2:**
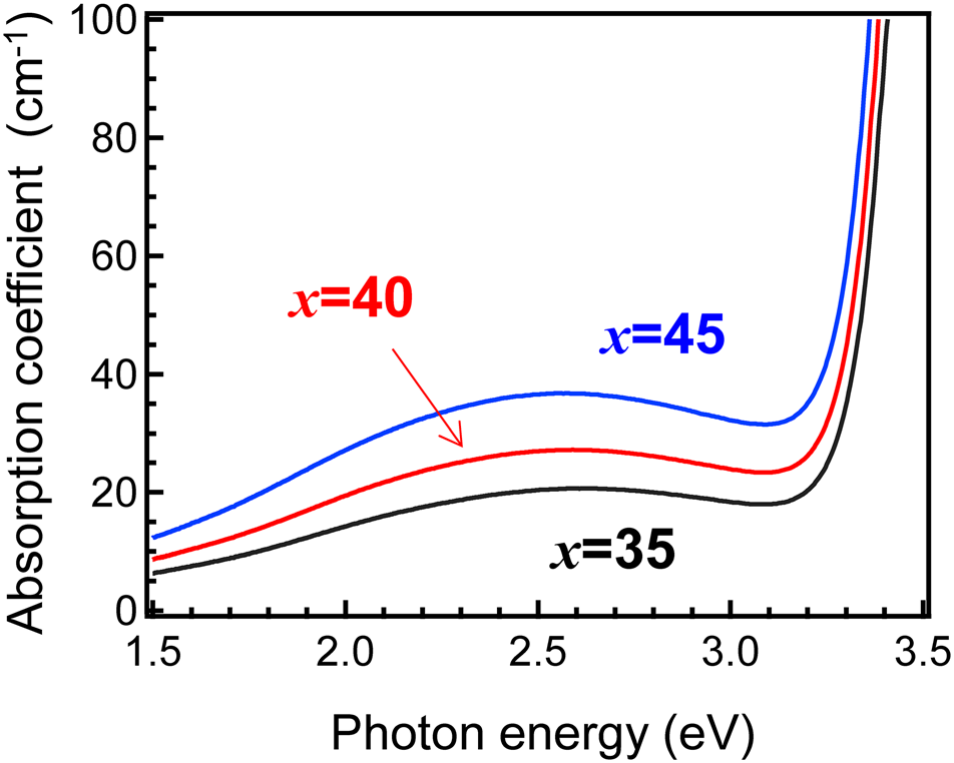
Optical absorption spectra of MTP*x* glasses.

The obtained MTP*x* glasses were brownish, as the colouration evolved with an increase in the TiO_2_ fraction. Figure 2 shows the optical absorption spectra of the MTP*x* glasses containing different TiO_2_ fractions. The optical absorption edge existed at approximately 3.3 eV, corresponding to the band gap of anatase^34^. In addition, there was an optical absorption band at 2.5 eV, due to the Ti^3+^ species^35^. The absorption coefficient linearly increased with increasing TiO_2_ fraction. Therefore, it was expected that the Ti^3+^:(Ti^3+^  + Ti^4+^) ratio would be approximately constant and independent of the chemical composition.

**Figure 3:**
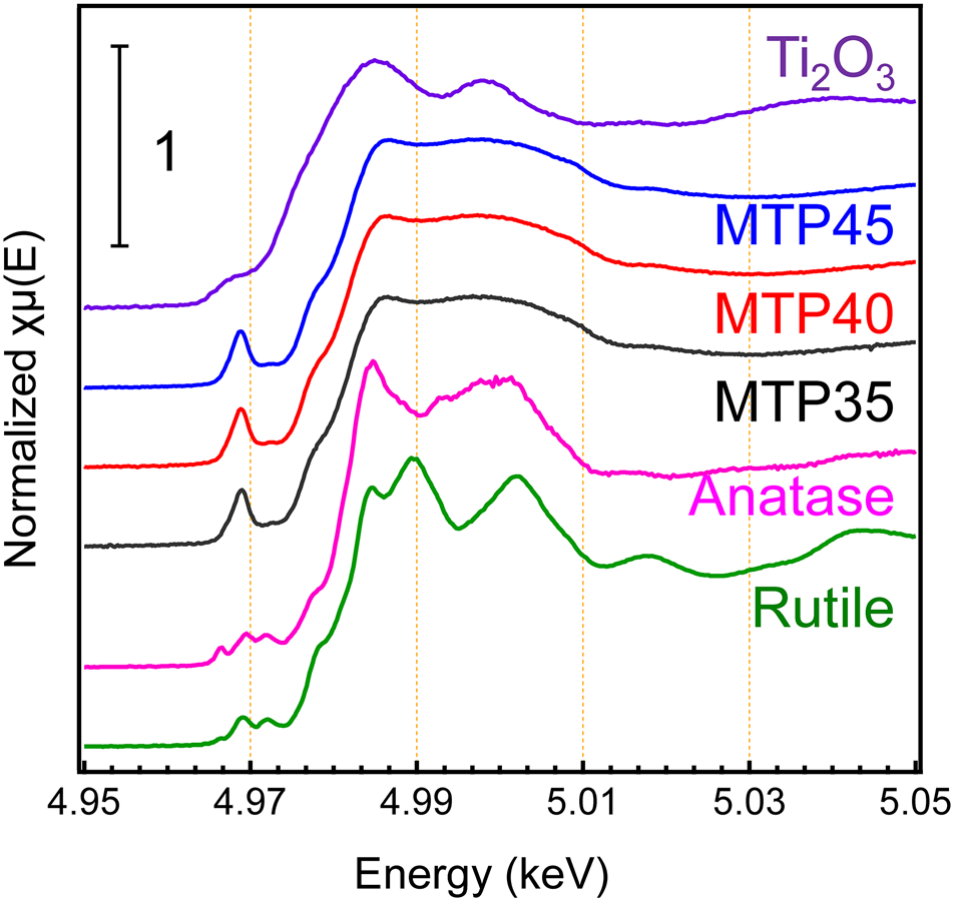
Comparison of the Ti K-edge XANES spectra: Ti K-edge XANES spectra of MTP*x* glasses with references—Ti_2_O_3_, rutile, and anatase.

To examine the valence state of the Ti cation, X-ray absorption near edge structure (XANES) was measured. Figure 3 shows the Ti K-edge XANES spectra of the MTP*x* glasses with Ti_2_O_3_, anatase and rutile. Compared with these references, it was assumed that the main valence state of Ti was tetravalent and was independent of the TiO_2_ fraction. Although the existence of Ti^3+^ was confirmed by the optical absorption, the concentration was not high enough to appear in the K-edge XANES spectra. It was notable that the spectral shapes of Ti in the MTP*x* glasses were similar to those of anatase. The similarity to anatase could be understood from the dip at 4.99 keV, although rutile TiO_2_ was used as a starting material. Thus, in the present MTP glasses, TiO_2_ existed with partial characteristics of anatase. Recently, it was reported that crystallization from a glass was affected by the local coordination of the mother glass, even though the chemical composition was not the stoichiometric chemical composition of a crystal^36^. As discussed below, it was found that the coordination of Ti in the mother glass affected the precipitated phase in the GCs.

**Figure 4:**
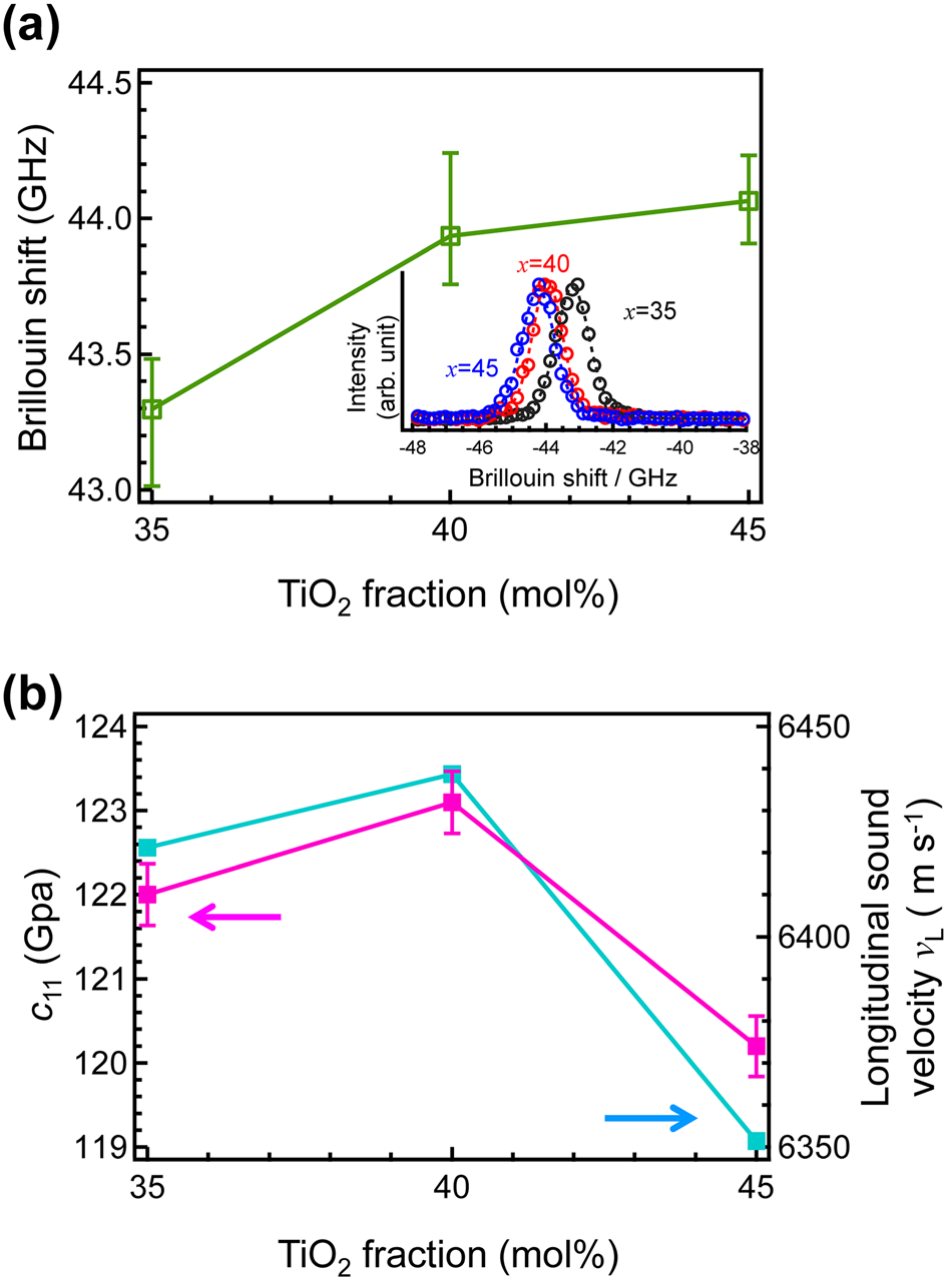
Mechanical properties of MTP*x* glass before crystallization: (**a**) Brillouin shift of MTP*x* glasses as a function of TiO_2_ fraction (inset shows Brillouin spectra of these glasses) and (**b**) longitudinal elastic modulus *c*_11_ and longitudinal sound velocity *v*_L_ of MTP*x* glasses as a function of TiO_2_ fraction.

Figure 4a shows the Brillouin shifts *ν*_B_ of the MTP*x* glasses obtained by fitting the Brillouin peaks, as shown in the inset. The density and the*ν*_B_ increased with increasing TiO_2_ fraction. On the contrary, the longitudinal sound velocity *v*_L_ and the longitudinal elastic modulus *c*_11_ indicated that MTP40 was the inflection point as shown in Fig. 4b. These parameters are summarized in Table 1. In the binary ZnO–P_2_O_5_ glass, the elastic modulus of the Zn-rich glass was higher than that of the ZnO-poor glass owing to the closed packed network consisting of ZnO_*x*_ polyhedral^37^. As TiO_2_ also belongs to the intermediate group in glass science, and can be assumed as either glass network-forming or network-modifying^38^, it was expected that the TiO_2_ network consisting of TiO_6_ anatase-like structure would be formed in TiO_2_-rich glasses. Although the first coordination states of Ti^4+^ cation are similar in these glasses, as shown in Fig. 3, the inflection point at the MTP40 suggested that the network structure had been changed. Therefore, the network consisting of TiO_6_ polyhedral and PO_4_ tetrahedron are discussed for PO_4_ units using ^31^P MAS NMR measurement.

**Table 1:** Physical properties of MTP*x* glasses.

	MTP35	MTP40	MTP45
*T*_g_ / °C	643	638	641
*T*_x_ / °C	765	748	758
*T*_p_ / °C	802	797	803
Density / g⋅cm^-3^ (error bars: ± 0.02)	2.96	2.97	2.98
Molar volume / cm^3^·mol^-1^	28.6	29.2	29.7
Refractive index at 532 nm	1.7935	1.8151	1.8455
Longitudinal sound velocity / m·s^−1^	6,421.2	6,438.7	6,351.4
*c*_11_ / Gpa	122.1	123.1	120.2

**Figure 5:**
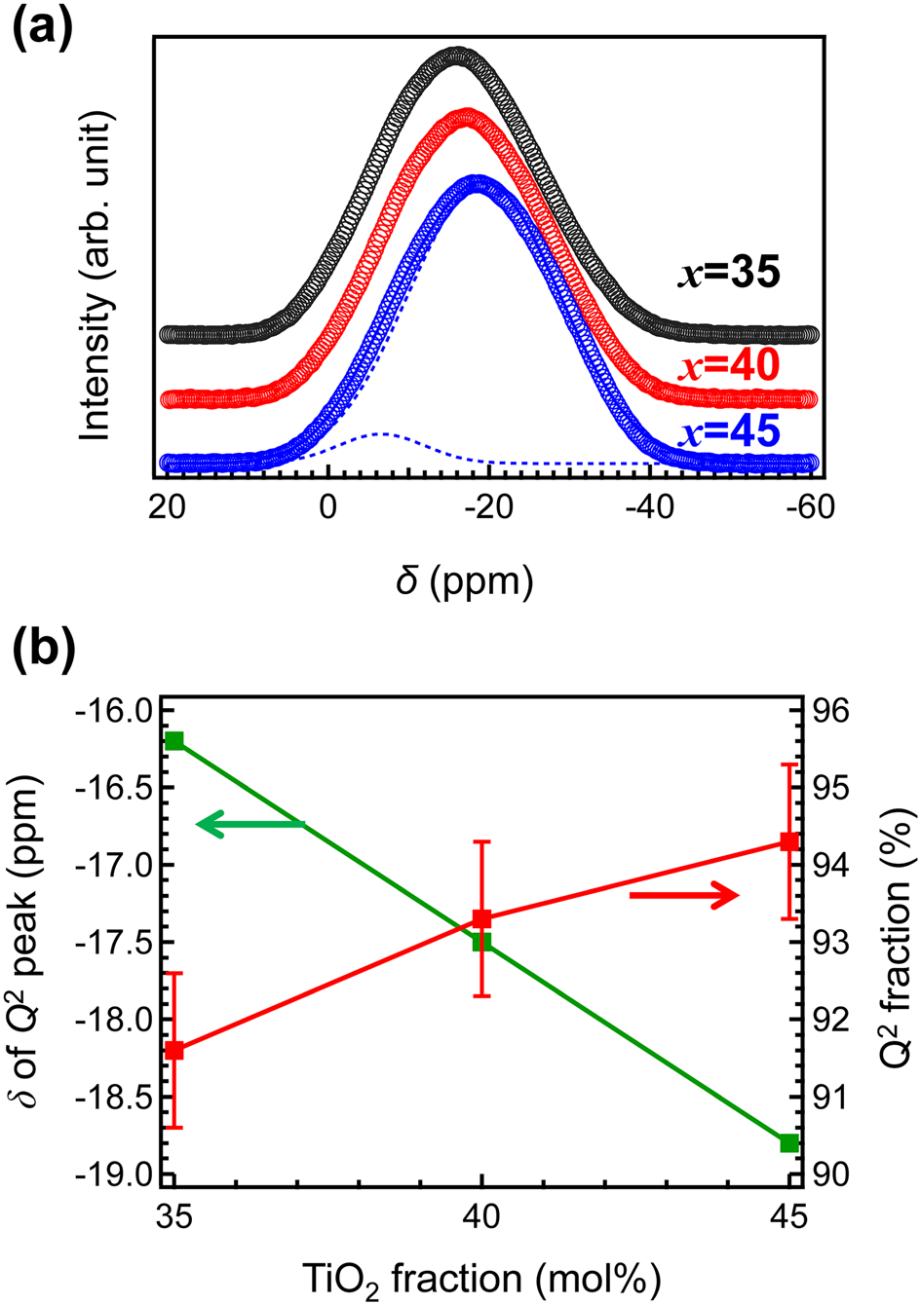
Structure of phosphorus network: ^31^P MAS NMR spectra of MTP*x* glasses (dashed lines indicate each *Q*^n^ fraction after peak deconvolution).

Figure 5 shows the ^31^P MAS NMR spectra of the MTP*x* glasses. In their study, the spectra were decomposed into different building units, denoted by *Q*^*n*^. These described the number of oxygen atoms (*n*) of the PO_4_ tetrahedral interlinked to other cations. Although P–O–Ti bonds were observed in the present NMR spectra, for simplicity, these observed peaks were assumed to have only covalent P-O-based bonds based on a previous study^39^. It is notable that the peak widths were constant, indicating that PO_4_ and TiO_6_ units were homogenously dispersed at the first and second coordination. According to previous studies^39–41^, MTP*x* glasses can be decomposed with individual signal components: *Q*^1^ and *Q*^2^, indicated with dashed curves. It was assumed that the slight chemical shift was due to the increase in the number density of the Ti–O–P bond, whose shielding was smaller than that of the P-O-P bonds. Therefore, no remarkable structural change in the PO_4_ units could be observed from the P MAS NMR spectra^31^.

**Figure 6:**
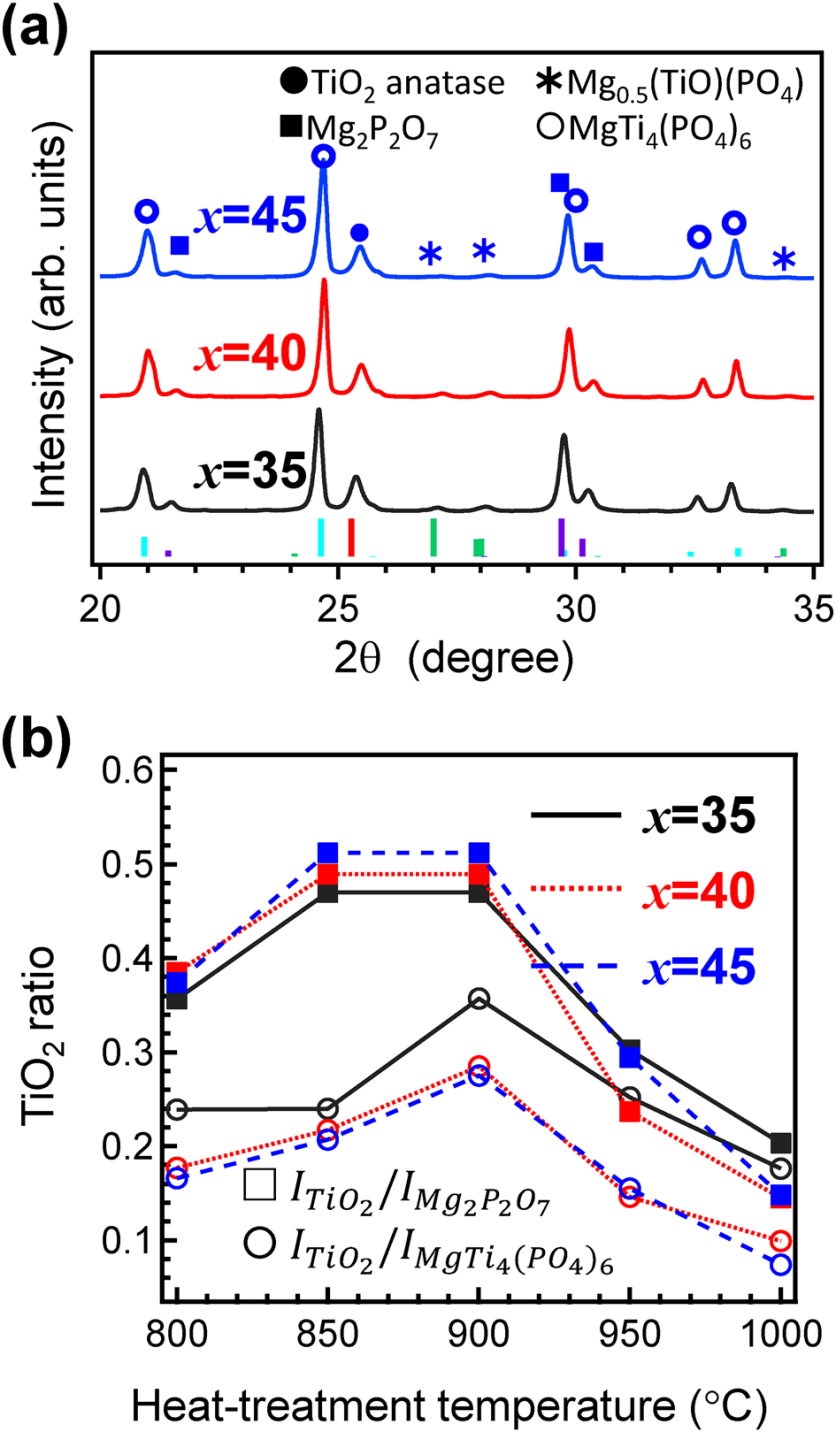
Crystallization behaviour of MTP*x* glasses: (**a**) powdered XRD patterns of the MTP*x* GCs after heat treatment at 900 °C for 3 h and (**b**) diffraction intensity ratios of $I_{{TiO_{2} }} /I_{{Mg_{2} P_{2} O_{7} }}$ and $I_{{TiO_{2} }} /$. $I_{{MgTi_{4} (PO_{4} )_{6} }}$ as functions of the heat treatment temperature.

Figure 6a shows the XRD patterns of powdered MTP*x* GCs heat-treated at 900 °C for 3 h with several references: TiO_2_ anatase, Mg_2_P_2_O_7,_ MgTi_4_ (PO_4_)_6_ and Mg_0.5_(TiO) (PO_4_). The obtained diffraction patterns showed that the anatase TiO_2_ crystallites co-precipitated with the other phases in all the GCs, and the precipitation of TiO_2_ as a single phase was not observed. It is often observed that metastable crystalline phase is precipitated after heat-treatment. Similar to previous results^28,29^, metastable anatase was precipitated from the present glass system. In addition, such co-precipitation of TiO_2_ and other phases was observed in the Na_2_O-doped CaO–TiO_2_–P_2_O_5_ GC systems^19^. To examine the relationship between the TiO_2_ precipitation and the heat-treatment temperature, the intensity ratio using the highest diffraction intensity was used for the evaluation. Figure 6b shows the TiO_2_ precipitation ratios, which were the relative intensities to the maximum diffraction intensities of Mg_2_P_2_O_7_ or MgTi_4_ (PO_4_)_6_, as a function of the heat-treatment temperature. The anatase TiO_2_ ratio achieved the maximum with heat-treatment at 900 °C, suggesting that an optimized heat-treatment temperature exists. The average particle diameters estimated from the Scherrer equation^42^ were approximately 27 nm.

**Figure 7:**
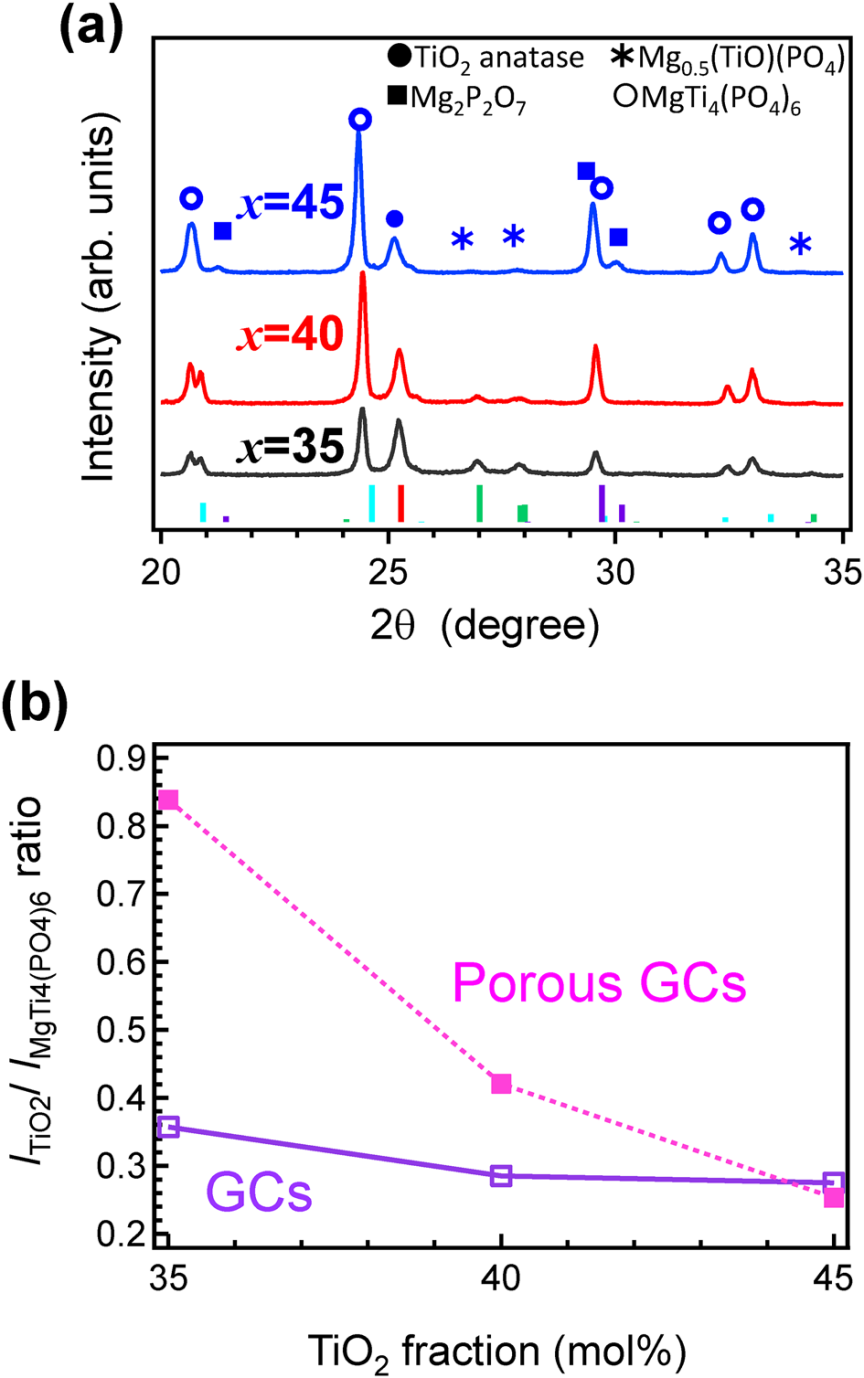
Effect of acid etching on the crystallized phase of samples: (**a**) powdered XRD patterns of the MTP*x* porous GCs obtained after acid leaching of GCs heat-treated at 900 °C for 3 h and (**b**) relative diffraction intensity ratios *I*_TiO2_:*I*_MgTi4(PO4)6_ as functions of the TiO_2_ fraction.

**Figure 8:**
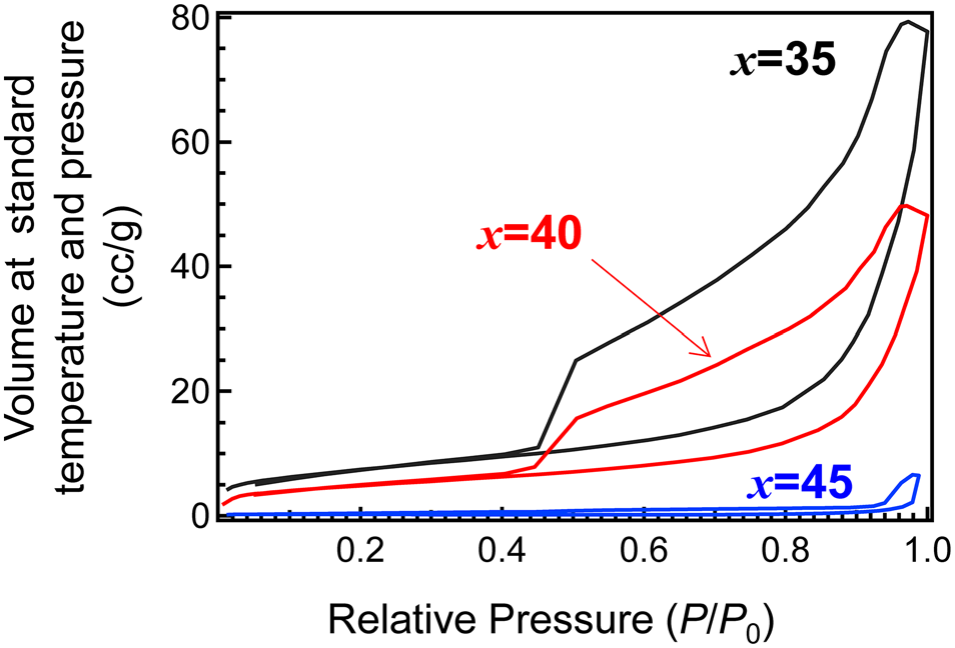
Volumes of adsorbed N_2_ at standard temperature and pressure of MTP*x* porous GCs as a function of relative pressure.

To obtain the porous GCs containing TiO_2_ crystallites, acid leaching of MTP*x* GCs heat-treated at 900 °C for 3 h using 1 M HNO_3_ was performed at 90 °C for 4 d. The results are summarized in Table 2. After acid leaching, the weight losses of MTP35, MTP40 and MTP45 glasses were calculated as 42 ± 5%, 31 ± 3%, and 8 ± 6%, respectively. This indicated that the chemical durability against acid leaching was enhanced with an increase in the TiO_2_ fraction. In addition, results of the eluted cations in the etched solution agreed with the results of the weight loss. This indicated that the TiO_2_-rich glass possessed a high chemical durability against acid solution. This narrowly correlated with a speculation based on the results of the elastic modulus (see Fig. 4). Figure 7a shows the XRD patterns of the MTP*x* porous GCs obtained after heat-treatment at 900 ºC for 3 h and their acid leaching. In acid-treated samples, the relative intensity of anatase increased compared with other phases, especially in the MTP35 system. Figure 7b shows the relative diffraction intensity ratio *I*_TiO2_:*I*_MgTi4(PO4)6_ as a function of TiO_2_ fraction. In the MTP35 system, the relative intensity of anatase increased compared with other systems. Crystallization of glass is a kind of thermally stabilization behavior of glass melt above the *T*_g_, and the precipitated crystalline phase and the residual amorphous region depend on the chemical composition. In the case of Mg-rich glass, it is expected that TiO_2_ can be precipitated easily because of better stability of residual Mg-rich phosphate amorphous region, which was easily removed by acid etching. Considering the results of weight loss shown in Table 2, it was assumed that several precipitated crystallites were removed due to acid leaching in the MTP35 system. Figure 8 shows the volumes of adsorbed N_2_ at standard temperature and pressure of MTP*x* porous GCs as a function of relative pressure. To understand the hysteresis shape, the vertical axis was plotted on a logarithmic scale. The hysteresis curves indicate the connected pores in the etched samples. Although the shapes of hysteresis curves of MTP35 and MTP40 were similar, the shape of MTP45 differed from the others. Additionally, the relative surface areas of porous MTP*x* GCs calculated from the Brunauer, Emmett and Teller (BET) method are shown in Table 2. The surface area decreased with increasing TiO_2_ fraction, and there was a large difference between the MTP40 and MTP45 porous GCs.

**Table 2:** Parameters after HNO_3_ leaching.

Sample ID	MTP35	MTP40	MTP45
Weight loss due to acid leaching / wt.%	42 ± 5	31 ± 3	8 ± 6
**ICP analysis in eluted HNO**_**3**_** solution (× 1/100)**			
Mg /ppm	24.6	18.4	3.97
Ti / ppm	0.007	0.011	0.083
P / ppm	28.4	16.3	2.26
**Surface area after leaching / m**^**2**^** g**^**−1**^			
Using BET method	26.5	17.3	1.01

**Figure 9:**
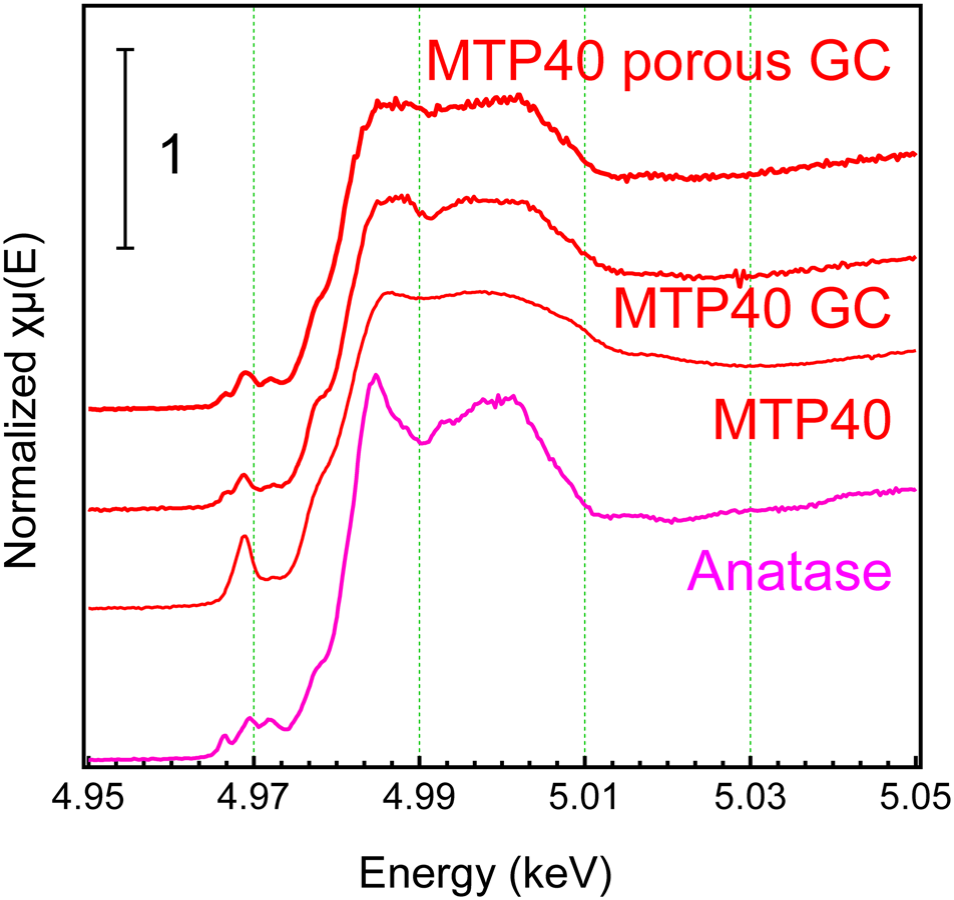
Structural change of Ti cations by XANES analysis: Ti K-edge XANES spectra of the MTP40 glass, GC, and porous ceramics after acid leaching, along with that of anatase.

Ti K-edge XANES spectra of the MTP40 glass, the GC and the porous GCs after acid leaching are shown in Fig. 9. In addition, the spectrum of anatase is shown for comparison. Compared with the mother glasses, characteristic absorption of anatase was clearly observed in the GC and the porous GC. Considering structural similarity, the anatase-like local structure in the glass network may have been the origin of the precipitation of the anatase TiO_2_ phase.

**Figure 10:**
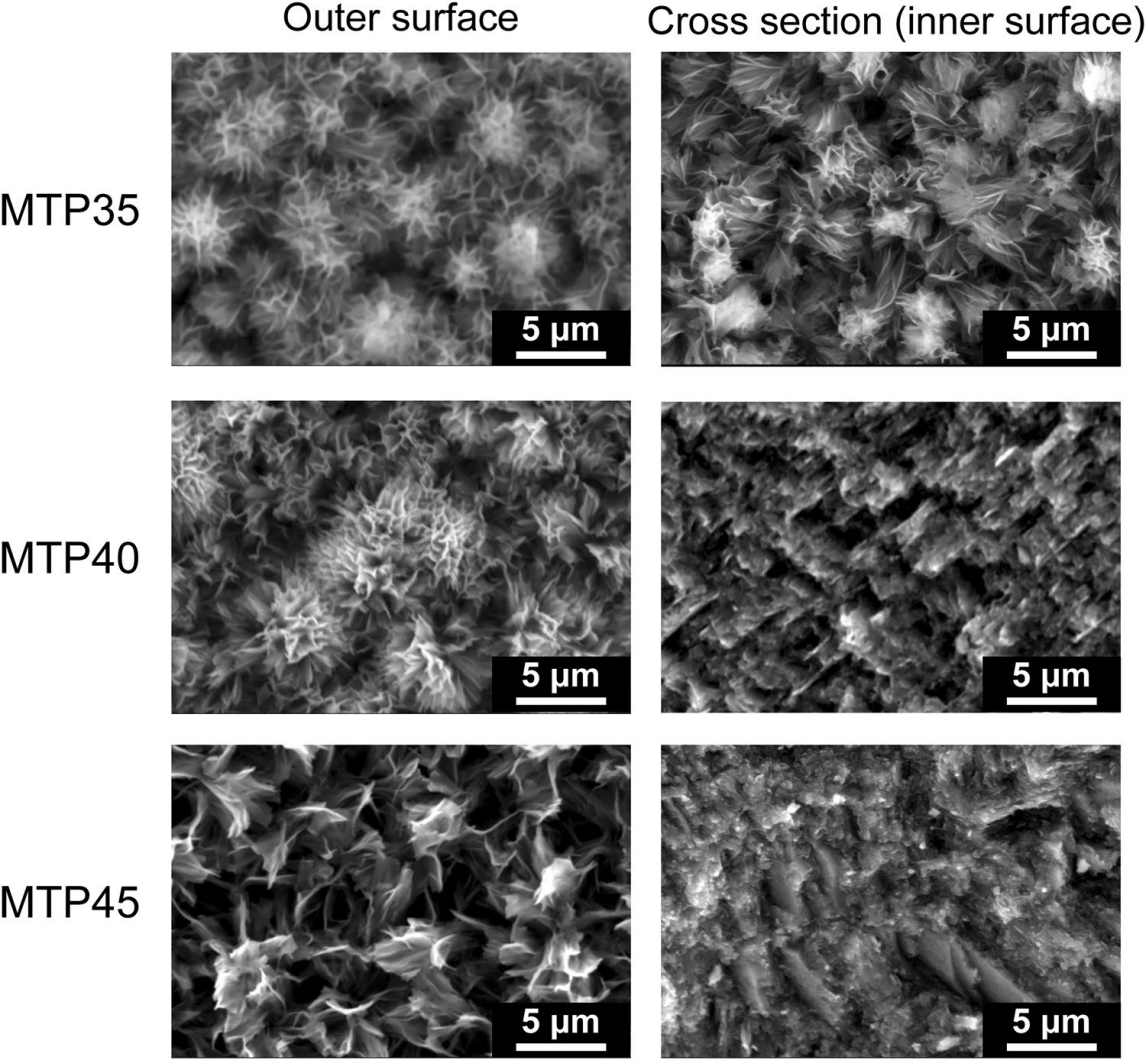
Morphology of the MTP*x* porous GCs after acid leaching: SEM images of outer surface and cross section of MTP35, MTP40, and MTP45 GCs after etching.

For discussion, the morphology of porous ceramics was checked after leaching by taking SEM images of the outer and inner surfaces. Figure 10 shows the SEM image of the MTP*x* porous GCs obtained after HNO_3_ (aq.) leaching of the GC heat-treated at 900 °C for 3 h. At the surface of the porous sample, dendrite-like structures were observed. Meanwhile, pillar-like structures were observed inside the MTP40 and the MTP45 porous ceramics, which was different from those of the MTP35 ceramics. Considering the values of *c*_11_, a change in the network structure affected the morphologies of the precipitated crystalline phases.

**Figure 11:**
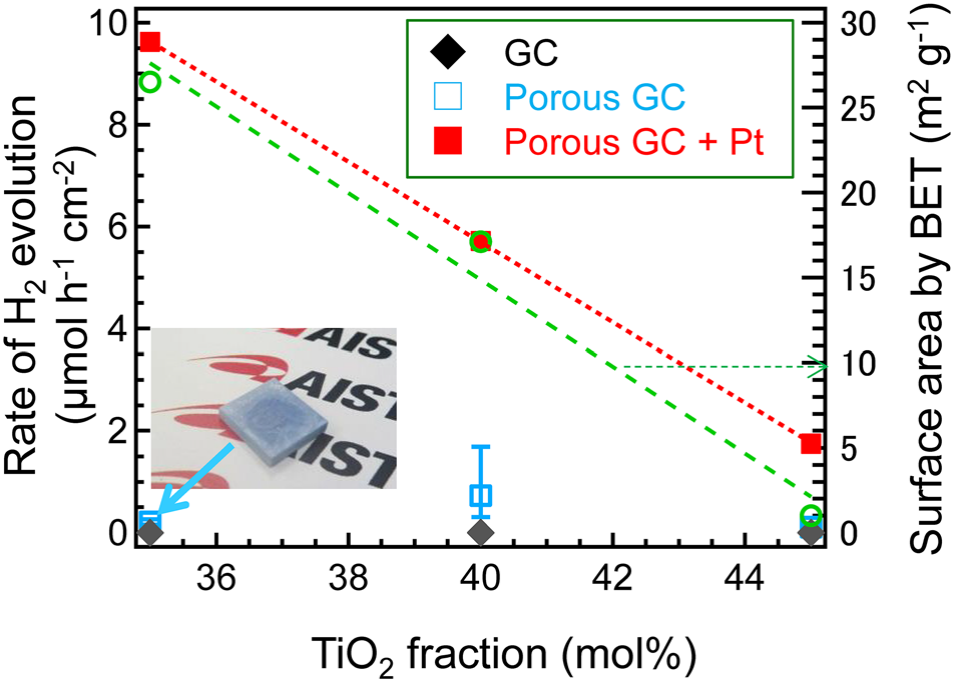
Hydrogen generation of the MTP*x* GCs: H_2_ evolution from the MTP*x* porous ceramics after acid leaching as a function of TiO_2_ fraction — BET surface areas of samples are shown using the right axis (inset is a photograph of the MTP35 porous GC after irradiation without Pt deposition).

**Figure 12:**
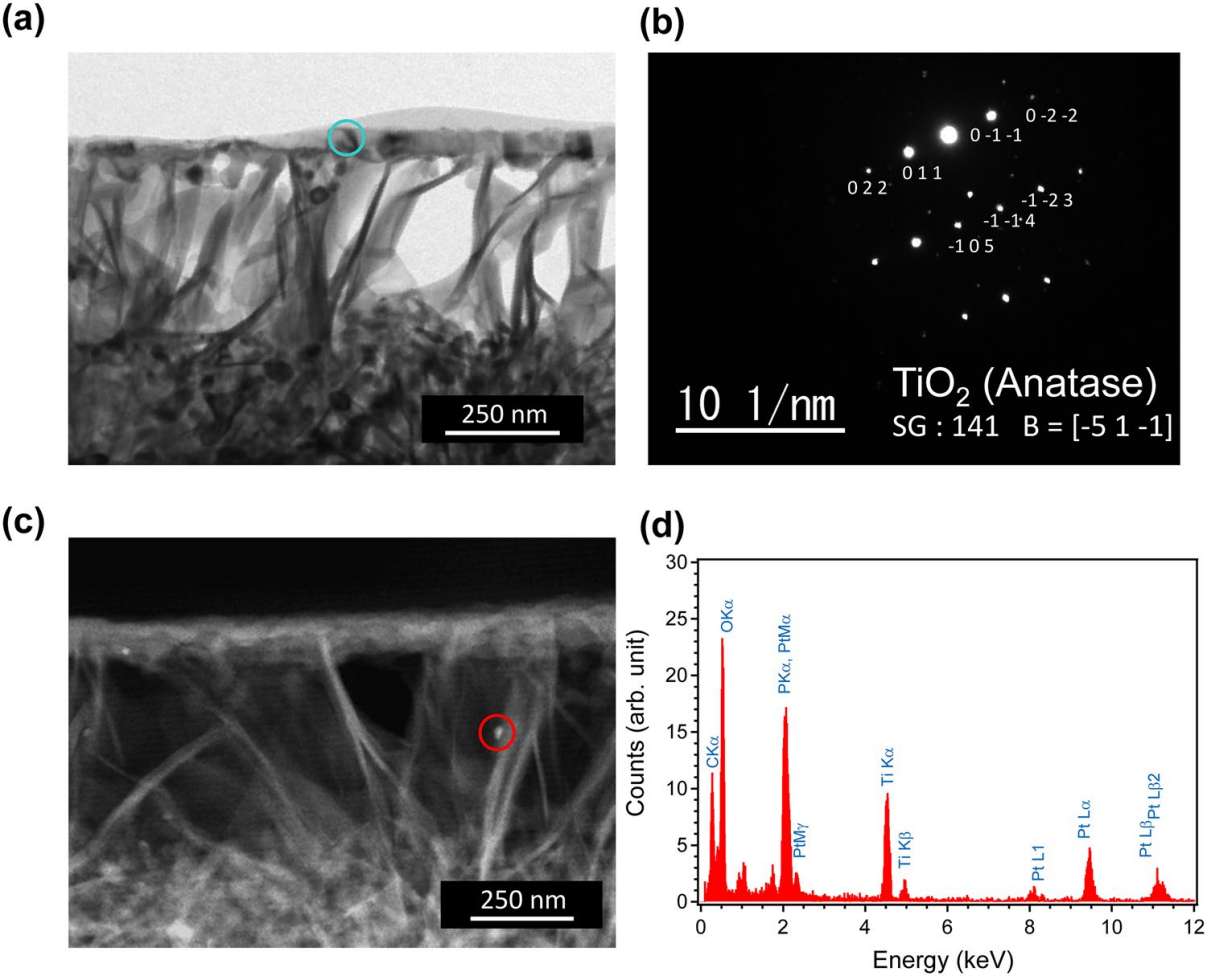
TEM observation of the porous MTP35 GC with Pt deposition: (**a**) cross-sectional bright TEM image of porous MTP35 GC with Pt deposition, (**b**) nanobeam electron diffraction pattern of MTP GC at the circled region in (**a**), (**c**) cross-sectional dark STEM image of porous MTP35 GC with Pt deposition, and (d) EDX profile of the circled region in (**c**).

Based on these results, the hydrogen evolution of the TiO_2_ GCs was examined using sacrificial molecules as electron donors for improvement of H_2_ production^43^. Figure 11 shows the hydrogen evolution rate from TiO_2_ GCs with and without acid leaching. Here, the H_2_ evolution rate per irradiated areas of the bulk samples (μmol⋅h^−1^⋅cm^−2^) are used to compare the photocatalytic activities of these monoliths. In the GCs without etching, the H_2_ evolution was approximately zero, independent of the chemical composition, indicating that the surface of GCs was covered with an amorphous region of low photocatalytic activity. To the contrary, the porous GCs exhibited H_2_ evolution activity without metal deposition^44–47^, with a maximum obtained with the MTP40 porous GCs. It should be noted that the relative surface areas of the porous GCs increased with decreasing TiO_2_ fraction (Table 2). The XRD patterns suggested that the relative intensity of precipitated anatase increased with decreasing TiO_2_ fraction. Therefore, from these results, it could be assumed that the MTP35 GCs should exhibit the highest photocatalytic activity. However, the result without metal deposition did not correspond to the expectation. It is notable that the sample surface turned blue in colour after the catalytic reaction, as shown in the inset of Fig. 11. As it was expected that the reduction in Ti(IV) to Ti (III) ^47,48^ generally inhibited catalytic performance, the generation of Ti(III) species should have been prevented, although an optimized Ti(III):Ti(IV) ratio is proposed^49^. In addition, to prevent the reduction reaction of Ti(IV), photocatalytic activity following Pt nanoparticle deposition was examined according to previous reports^45,46^. Notably, the photocatalytic activity of MTP35 was highly enhanced due to Pt deposition, as shown in Fig. 12. It was suggested that the H_2_ evolution rates broadly depended on the relative surface area. Thus, it was concluded that relative high surface area and prevention of the reduction reaction to Ti(III) were the most important factors in tailoring monolithic photocatalytic materials.

Here, we have compared the H_2_ generation rate of the present study with those of previous cases, as shown in Table 3. In Table 3, we only focused on the TiO_2_ GCs materials that could be treated as monolithic plates on the water. The present MTP GC exhibited the best H_2_ generation rate among the monolithic glass–ceramics that can be prepared to larger sizes. Although the present result was not optimized, there should be great potential for improving H_2_ generation or environmental cleaning properties using GC routes.

**Table 3:** Comparison of H_2_ production rates from TiO_2_ glass–ceramics.

Titania glass–ceramics photocatalyst	Form of the sample	BET surface area / m^2^ g^−1^	Reaction medium	Light source	Rated power / W	Rate of H_2_ production		Ref
						/ µmol h^-1^ cm^−2^	/ µmol h^-1^ g_cat._^−1^	
Pt/MTP35	etched glass (9 × 9 × 1 mm)	26.5	MeOH + H_2_O (1:1)	Hg lamp	100	9.6	35.7	This work
MTP40	etched glass (12 × 10 × 1 mm)	17.3	MeOH + H_2_O (1:1)	Hg lamp	100	0.73	5.7	This work
Pt/TZBAS	etched glass (30 × 30 × 1 mm)		MeOH + H_2_O (1:9)	Xe lamp (< 500 nm)	300	0.12		^30^
Pt/TBZA-BN	non-etched glass (30 × 30 × 1 mm)		MeOH + H_2_O (1:9)	Xe lamp (< 500 nm)	300	0.021		^31^
Pt/TBZA-BN	non-etched glass (30 × 30 × 1 mm)		MeOH + H_2_O (1:9)	Xe lamp (> 440 nm)	300	0.009		^31^
PTG	etched glass (5 mm Φ × 1 mm)	146	MeOH + 50% KOH	Xe lamp	2,500		4.0	^50^
Cu/PTGB	etched glass (5 mm Φ × 1 mm)		MeOH + 50% KOH	Xe lamp	2,500		187	^50^

MTP*x*: (70-*x*)MgO–*x*TiO_2_–30P_2_O_5_.

TZBAS: 14TiO_2_–23ZnO–45B_2_O_3_–18Al_2_O_3_–4.5SiO_2_.

TBZA-BN: 20TiO_2_–45B_2_O_3_–45ZnO–20Al_2_O_3_–20BN.

*PTG* porous titania glass.

*PTGB* porous titania glass treated with base.

To understand the hydrogen generation rates and the structure at the surface, a transmission electron microscopy (TEM) image was taken of the porous MTP35 GC after acid etching with Pt deposition. Figure 12a shows a cross-sectional bright TEM image of the porous MTP35 GC with Pt deposition. At the surface, there are small islands in addition to fibrous pillar structure. This is also confirmed in Fig. 10. The precipitation of anatase at the surface was also confirmed from the nanobeam electron diffraction pattern shown in Fig. 12b (the morphology on a larger scale is shown in the Supplemental Information). Because Pt nanoparticles are difficult to observe in the bright TEM image, a dark scanning TEM (STEM) image of the porous MTP35 GC was also taken, as shown in Fig. 12c. In the dark STEM image, heavy elements can be detected as bright spots. One can observe white spots both at the surface (at the left side of the figure) and at the fibrous pillar region. The energy-dispersive X-ray spectroscopy (EDX) profile of the white spot — circled region in Fig. 12c—is shown in Fig. 12d. The profile shows that Pt particles with diameters of approximately 5 nm are randomly dispersed. However, it was confirmed that fibrous pillar-like structures consist of several crystalline phases (see the Supplementary Information). Therefore, the sparse structure at the surface, which consisted of anatase regions and fibrous pillars, affects the hydrogen generation. It has been reported that the morphology of the sample also contributes to the H_2_ production, because the interior microporous channels provide an easy path for the electrons for the effective surface charge transfer^51^. In principle, photocatalytic activity increases with increasing surface area. If the surface areas of these samples were equal, the MTP45 with the highest TiO_2_ fraction might exhibit the best performance. However, in the present case, there is an inverse relationship between the relative surface area of porous GCs and the TiO_2_ fraction of the samples. Since the porous MTP35 GC has the highest surface area (see Fig. 8), we conclude that photocatalytic activity of the porous MTP GCs are dominated by the relative surface area. Because nanostructures at the surface are important to enhance the specific surface area, the glass–ceramic route combined with chemical etching is important to attain high photocatalytic activity. Although the present materials are not the optimized photocatalytic materials, it was demonstrated that monolithic bulk materials have the potential to be catalytic materials that can contribute to a sustainable society.

To summarize, photocatalytic activity has been demonstrated with and without Pt nanoparticle deposition. The Pt deposition is effective for enhancement of the catalytic activity as it prevents a reduction in TiO_2_. For application in sustainable energy conversion using porous TiO_2_-precipitated materials, tailoring the TiO_2_ crystallites in addition to the skeleton network is required. However, as the present materials are monolithic and effective for surface tailoring using the vapour reaction, further improvement using surface treatment is required^52,53^. To obtain porous GCs with good catalytic activity, the precipitated morphology of crystallites along with the nature of residual amorphous phases should be considered during materials design. For materials design, an understanding of the nucleation and crystal growth processes in glass is necessary, and structural analysis combined with several different measurement techniques will be helpful in this regard. For better formability, this study emphasizes that porous GC will be a candidate for energy harvesting following further performance improvements.

## Methods

### Preparation of MgO–TiO_2_–P_2_O_5_ glasses and GCs

The (70-*x*)MgO–*x*TiO_2_–30P_2_O_5_ (MTP*x*) precursor glass was prepared using a conventional melt-quenching method. Batches consisting of MgO (99.9%), TiO_2_ (rutile, 99.9%) and (NH_4_) H_2_PO_4_ (99%) were mixed and calcined at 800 °C for 3 h in open air, in order to prevent damage of Pt crucibles^54^. The obtained solids after calcination were then melted in a platinum crucible in an electric furnace at 1,300 °C for 30 min. The glass melt was quenched on a stainless steel plate at 180 °C, then annealed at the temperature of glass transition, *T*_*g*_, for 30 min. After mechanical polishing to obtain a mirrorlike surface, the glass sample was heat-treated on an alumina plate in an ambient atmosphere to obtain the corresponding GCs. The heat-treatment strategy consisted of three steps. The heating rate was 10 °C/min from room temperature (RT) to 30 °C below the target temperature, and then reduced to 1 °C/min to the target temperature. After heat treatment at the holding temperature for 5 h, the furnace was cooled down without temperature control.

### Preparation of porous GCs

The GCs were leached in 1 M HNO_3_ (50 mL) at 90 °C for 4 d without stirring. After leaching, the samples were rinsed with pure water at 90 °C for 2 h, then rinsed in an ultrasonic bath using pure water for approximately 10 min. After rinsing, the samples were heated at 150 °C for 2 h to obtain the porous GCs.

### Analysis methods

The *T*_*g*_, crystallization onset, *T*_*x*_ and the crystallization peak *T*_*p*_ were measured using differential thermal analysis (DTA) operated at a heating rate of 10 °C/min using TG8120 (Rigaku). The densities were measured by applying the Archimedes method using water at RT. The refractive indices (error bars ± 0.0001) at 452 nm, 633 nm, and 832 nm were measured using a prism coupler (Metricon, NJ). The refractive indices at 532 nm were calculated using lambda-in-Cauchy fitting [*A* + *B*/(*l*^2^) + *C*/(*l*^4^)] of these three values. Here, *l* was the wavelength, and *A*, *B* and *C* were the fitting parameters. The Brillouin shifts, *ν*_B_, of the glasses were measured using a high-resolution modification of a Sandercock–Fabry–Perot system^55^. The longitudinal sound velocity, *V*_L_, was calculated using the equation *V*_L_ = *ν*_B_*λ*/2*n*_532_, where *ν*_B_, *λ* and *n*_532_, are the Brillouin shift, the wavelength of incident light (532 nm), and the refractive index at 532 nm, respectively. The *c*_11_ values were calculated using the equation *c*_11_ = *ρV*_L_^2^, where *ρ* is the density. The absorption spectra were measured with a spectrometer UV-4150 (Hitachi High-Tech). X-ray diffraction (XRD) using UltimaIV (Rigaku) was used for examining the precipitated phases.

The ^31^P NMR of the precursor glasses and the GCs were measured using DELTA (JEOL) under 14.1 T. A frequency of 161.80 MHz, a spin rate of 10 kHz and a pulse delay of 5 s were used in the measurements. The chemical shifts were estimated with respect to 85% H_3_PO_4_ aqua solution (0 ppm) and the conventional notation for phosphorus sites, *Q*^*n*^, was used for the analysis. The *n* value denotes the number of bridging oxygen atoms per PO_4_ tetrahedron.

The Ti K-edge (4.98 keV) XANES spectra were measured at the BL01B1 and BL14B2 beamlines of the SPring-8 synchrotron radiation facility (Hyogo, Japan). The measurements were performed using a Si (111) double-crystal monochromator in the transmission mode (Quick Scan method) at RT. Pellet samples for the measurements were prepared by mixing the granular sample with boron nitride. The corresponding analyses were performed using Athena software^56^**.**

To examine the durability against acid solutions, a sample weight was measured before and after 1 N HNO_3_ leaching. In addition, Inductively Coupled Plasma-Atomic Emission Spectrometry (ICP-AES) measurement of the leaching solution was performed using SPS7800 (Hitachi High-Tech) to check the eluted composition. The surface areas of the samples were measured using NOVA3200 (Quantachrome). The morphology of the GCs was measured using a scanning electron microscope (SEM), where SEM images were taken using a JSM-6510 (JEOL). An HF-2000 (Hitachi) microscope was used to obtain TEM images and perform EDX with an acceleration voltage of 200 kV.

### Photocatalytic hydrogen evolution from methanol aqueous solution

A gas chromatography vial equipped with an open-top screw cap bottle sealed with a butyl rubber septum (SVG-12, Nichiden-Rika Glass Co. Ltd., internal volume of 15.6 mL) was employed as the reaction vessel. A GC sample was placed at the bottom, and a 50 vol.% aqueous solution of methanol (5 mL) was added. Nitrogen gas was bubbled into the solution for 30 min to remove dissolved oxygen. The reactor was irradiated from bottom with a 100 W high pressure Hg lamp (HL100G, SEN Lights Corp.) and cooled using a fan. The irradiation intensity was 342 mW/cm^2^. The headspace gas was sampled (0.2 mL) with a gas-tight syringe at 20 min intervals and analysed using a TCD gas chromatograph (GC320, GL Sciences Co., Ltd.) equipped with a MS5A column (2 m) of flowing Ar as the carrier gas^57^.

### Platinum deposition on the surface of glass–ceramics samples

Spontaneous reduction of platinum ions on the surface of the glass–ceramics sample was performed in accordance with the reported method^45,46^ using the sample with oxygen vacancies obtained after photocatalytic hydrogen evolution. After the photocatalytic reaction, the bluish-gray glass–ceramics sample was washed with distilled water several times. A H_2_PtCl_6_ (10 mM) aqueous solution of 0.31 mL was added to 5.0 mL of methanol (50 vol. %) aqueous solution. The glass–ceramics sample with oxygen vacancies was added to the solution, and was left to stand for 15 min with occasional stirring. The resulting sample with Pt deposition was washed, and used again for photocatalytic H_2_ evolution.

## Supplementary information

Supplementary file1 (PDF 2424 kb)

## Data Availability

The datasets generated and/or analysed during the current study are available from the corresponding author on reasonable request.
