# Supplementary material for: Photocatalytic hydrogen generation of monolithic porous titanium oxide-based glass–ceramics
Source: Sci Rep. 2020 Jul 15;10:11615. doi: 10.1038/s41598-020-68410-7 (PMC7363924; doi:10.1038/s41598-020-68410-7)
Supplement: Supplementary file 1 — Supplementary file1 (PDF 2424 kb) [file 41598_2020_68410_MOESM1_ESM.pdf]

Supplementary Information

Photocatalytic Hydrogen Generation of Monolithic Porous Titanium Oxide-based Glass–Ceramics

Hirokazu Masai, Hiroaki Sakurai, Akitoshi Koreeda, Yasuhiro Fujii, Takahiro Ohkubo, Takamichi Miyazaki, and Tomoko Akai

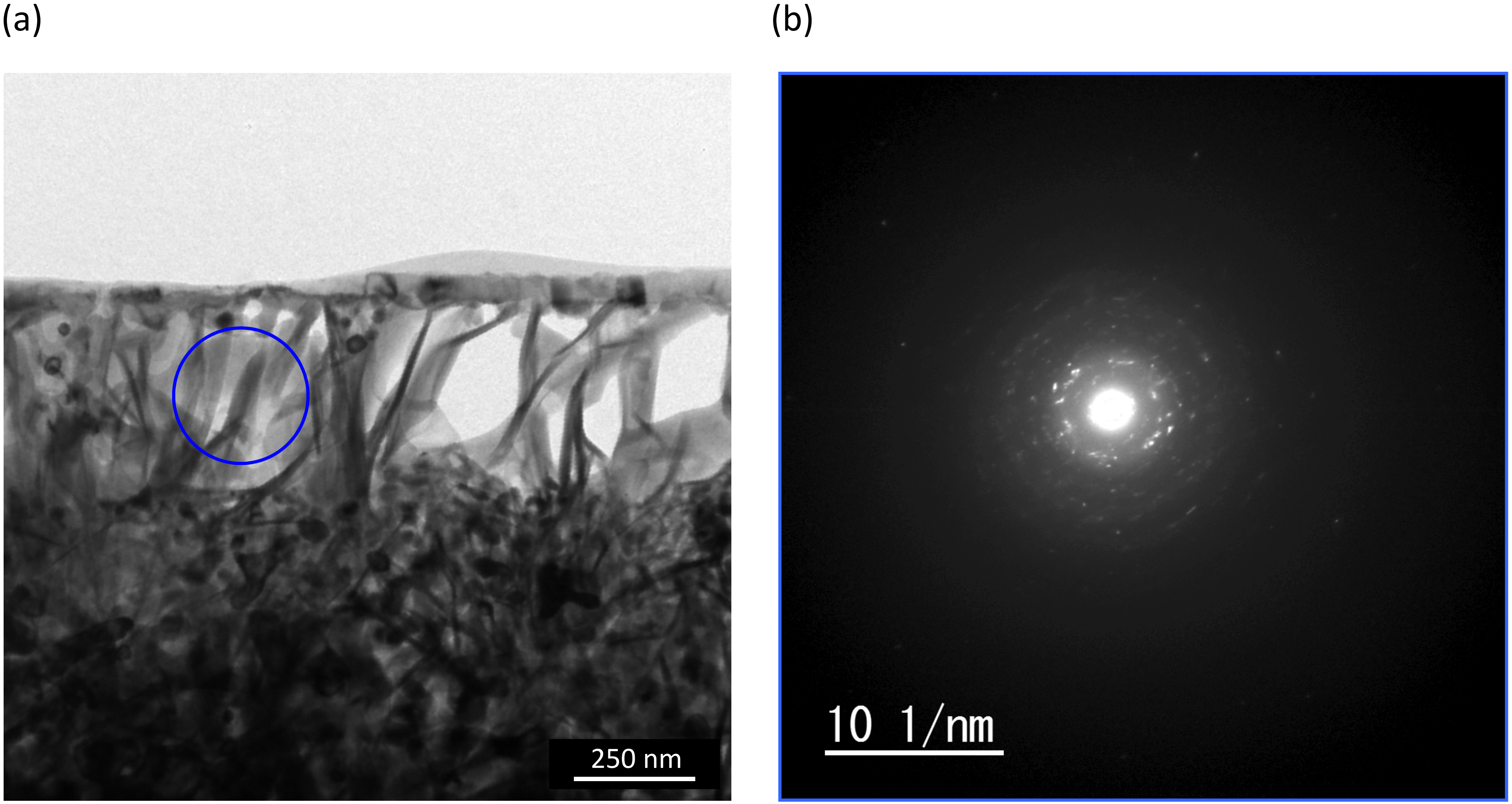

Supplemental Figure 1

(a) Cross-sectional bright TEM image at the needle-shaped region and (b) nanobeam electron diffraction pattern at the circled region in supplemental Fig. 1(a).

(a) (b)

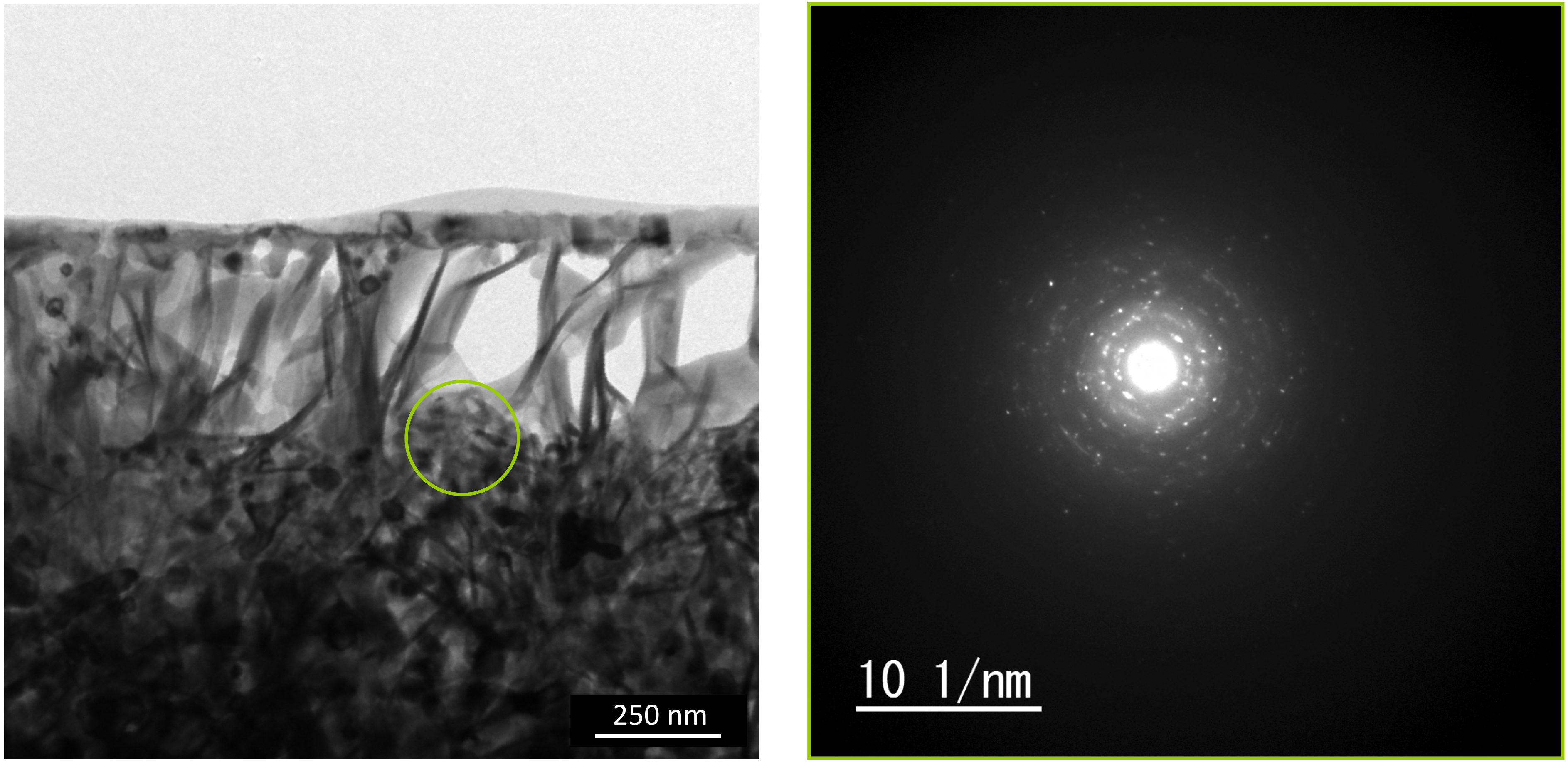

Supplemental Figure 2

(a) Cross-sectional bright TEM image at the needle-shaped region and (b) nanobeam electron diffraction pattern at the circled region in supplemental Fig. 2(a).

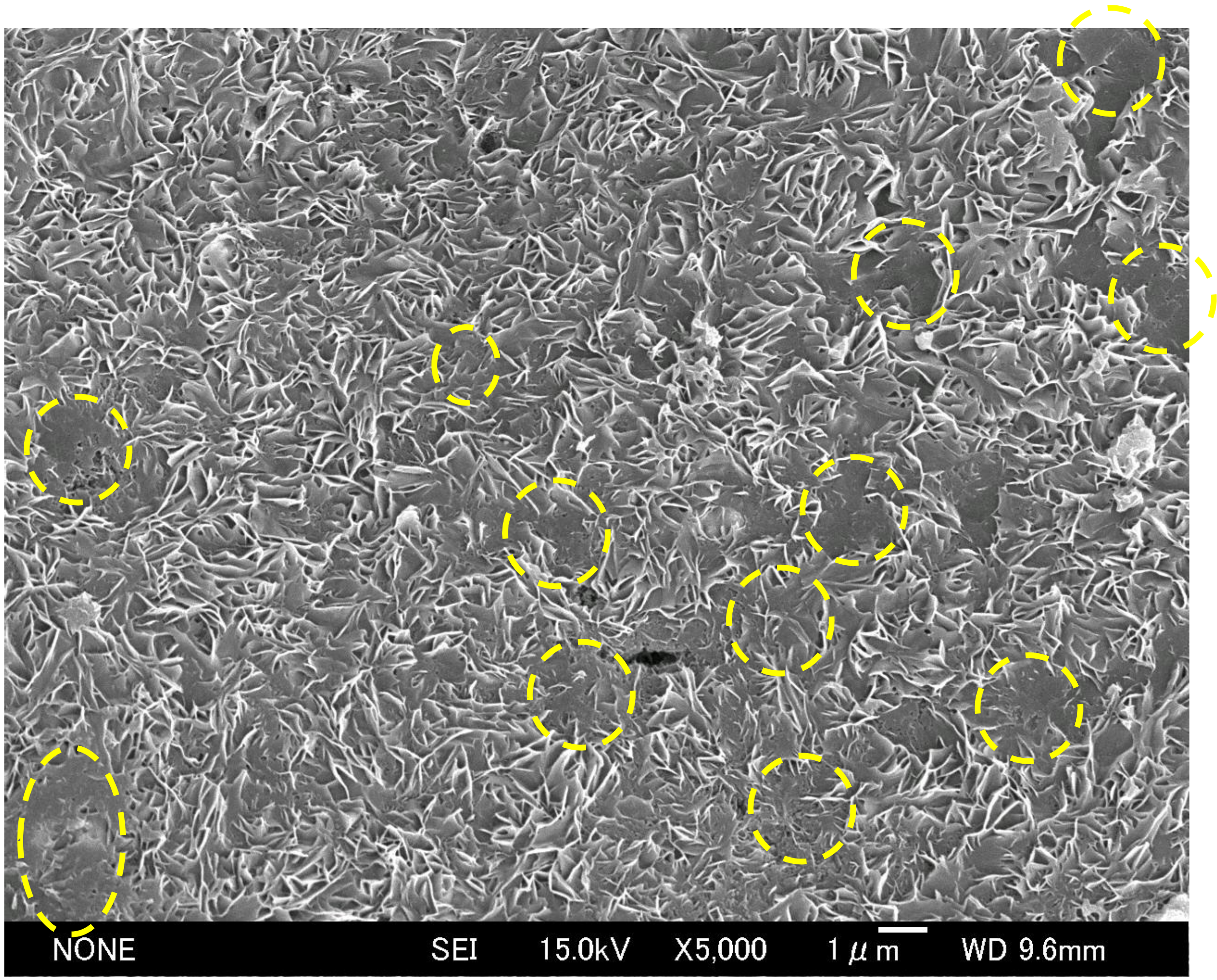

Supplemental Figure 3

SEM image of the MTP35 GC after acid etching: this image was taken using JSM-7100F (JEOL) — dotted circles indicate the anatase-rich surface regions, whose cross section is shown in Fig. 12(a).

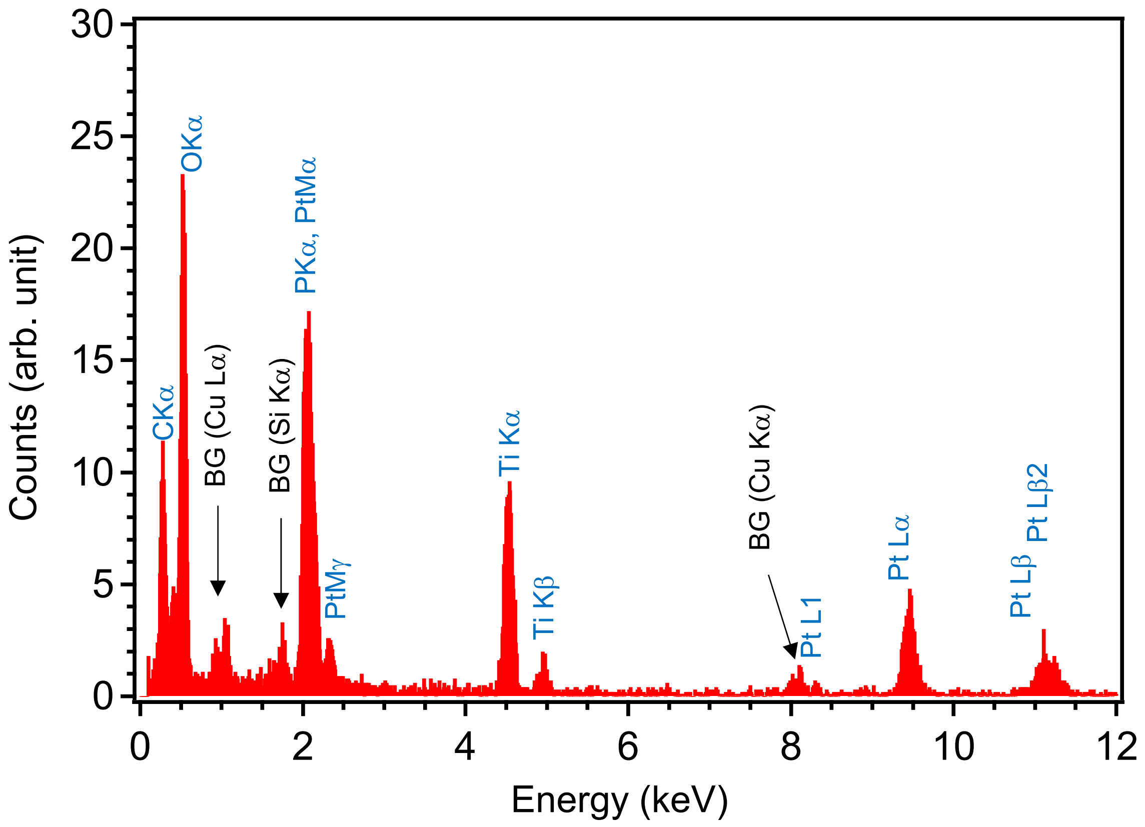

Supplemental Figure 4

EDX profile of the circled region in Fig. 12(c) along with the background (BG): BG peaks consist of the mount material (Si wafer) and Cu mesh.
